# Functional opposition between habenula metabolism and the brain reward system

**DOI:** 10.3389/fnhum.2013.00662

**Published:** 2013-10-10

**Authors:** Jason Shumake, F. Gonzalez-Lima

**Affiliations:** Department of Psychology, University of Texas at Austin, AustinTX, USA

**Keywords:** habenula, reward, brain metabolism, depression, emotion

Matsumoto and Hikosaka (2007) provided the first electrophysiological evidence that lateral habenula (LHb) neurons are inhibited by stimuli predicting reward and excited by stimuli predicting no reward, in exact opposition to the firing responses of midbrain dopamine (DA) neurons. Discovering the temporal relationship between LHb neuronal activity and negative reward prediction was groundbreaking. But for the metabolic brain-mapping field, development of a more general model of functional opposition between the LHb and the brain reward system (BRS) began with metabolic findings in the 1980s (Shumake and Gonzalez-Lima, 2003).

## 2-Deoxyglucose (2-DG) studies of LHb function in the 1980s

Louis Sokoloff and colleagues were the first to demonstrate that DA manipulations profoundly influence metabolism in the LHb. Specifically, amphetamine and apomorphine, drugs which increase DA levels and activate DA receptors, respectively, markedly decreased LHb glucose consumption; in contrast, haloperidol, which blocks DA receptors, markedly enhanced glucose utilization by the LHb (Wechsler et al., 1979; McCulloch et al., 1980). Rewarding electrical stimulation of the medial forebrain bundle (MFB) also suppressed LHb metabolism, whereas doses of the DA-antagonist pimozide both abolished self-stimulation of the MFB and caused dramatic elevations in LHb metabolism (Gomita and Gallistel, 1982; Gallistel et al., 1985).

Decreased LHb metabolism in response to increased DA levels was further demonstrated by Porrino and colleagues, who found a strong inverse relationship between LHb glucose uptake and both the dose of DA-increasing drugs and their associated locomotor effects (Porrino and Lucignani, 1987; Porrino et al., 1988). Increased LHb metabolism was further demonstrated in response to an array of DA-receptor blockers (Ramm et al., 1984), catecholamine depletion with alpha-methyl-para-tyrosine (AMPT), and amphetamine withdrawal (Caldecott-Hazard et al., 1988). Because amphetamine suppresses LHb metabolism when administered acutely, elevated LHb metabolism during amphetamine withdrawal implies that the LHb develops a compensatory response against chronic drug administration, rebounding in the opposite direction when treatment is withdrawn.

Whereas most of the 2-DG studies during this time period relied on pharmacological manipulations to effect change in LHb metabolism, Gonzalez-Lima and Scheich (1986) were the first to link changes in LHb activity to learning. They found that LHb metabolism was selectively increased by contiguous pairings of a conditioned stimulus (CS) with an aversive unconditioned stimulus (US). Since the LHb was not activated by presentations of the CS alone or the US alone, this implied that the LHb responded to the *prediction* of the US by the CS. This finding not only anticipated the discovery of the negative-reward predicting properties of LHb neurons, but also suggested that LHb neurons may be involved in the prediction of aversive events more generally, i.e., the withdrawal of a pleasant stimulus or the delivery of an unpleasant one.

## Reciprocal inhibition and bistable states

**Figure 1:**
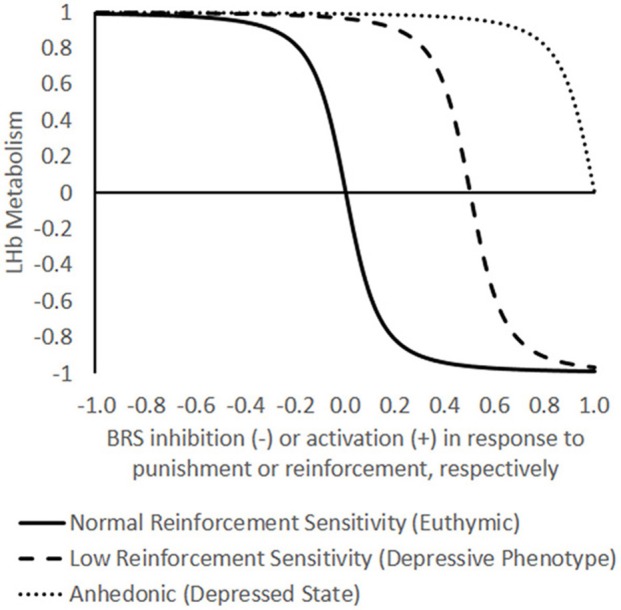
**Theoretical model of the functional opposition between lateral habenula (LHb) energy metabolism and the brain reward system (BRS).** In the model, LHb metabolism (Y-axis) may oscillate between a neutral baseline (0) to maximal metabolism (+1) and minimal metabolism (−1). The X-axis represents BRS inhibition or activation in response to an emotional event that ranges from −1, the maximum negative reward/punishment (withdrawal of pleasant stimulus or infliction of unpleasant stimulus) to +1, the maximum reward/negative reinforcement (delivery of pleasant stimulus or withdrawal of unpleasant stimulus). Note that the choice of axes is arbitrary in terms of independent and dependent variables; in reality, the BRS and LHb are mutually interdependent. Differences in reinforcement sensitivity are represented by three separate curves (normal, low, and anhedonic) defined by the function $-\frac{A+E}{\sqrt{{\left(A+E\right)}^{2}+B^{-1}}}$ where *A* is the preexisting affective state, *E* is an emotional event represented on the X-axis, and *B* is the degree of bistability (the steepness of the transition between high and low energetic states), held constant here at an arbitrary value of 50. The model captures the dramatic, bimodal shifts in LHb metabolism that have been empirically observed in response to manipulating the brain reward system (BRS) in “euthymic” animals with normal reward sensitivity (*A* = 0). High energy states of the LHb (positive values on Y-axis) are presumed to inhibit the BRS, and low energy states (negative values on Y-axis) are presumed to disinhibit it. The model predicts that increasing levels of depressive predisposition × environmental stress (negative affect, *A* < 0) are reflected by increasing rightward shifts in the reinforcement-response curves such that increasing levels of reinforcement frequency or intensity are needed to bring the LHb into a low energy state until eventually a state of anhedonia is reached in which no amount of reinforcement is sufficient. The model predicts that differences in LHb function between depressives and healthy controls are more likely to be revealed under conditions of rewarding feedback (diverging curves on right side of graph) than under punitive feedback (converging curves on left side of graph).

In general, then, the LHb and the BRS display a mutually inhibitory relationship in terms of functional connectivity. This does not mean, for example, that the LHb and VTA send inhibitory *anatomical* projections directly to one another. The major anatomical pathway of influence to the VTA from the LHb appears to be an excitatory projection to the rostromedial tegmental nucleus, which then sends an inhibitory projection to the VTA (Balcita-Pedicino et al., 2011; Hong et al., 2011). Nevertheless, the *functional* relationship between the LHb and BRS can be modeled as a circuit of reciprocal inhibition. Based on other models of reciprocal inhibition between other neuronal populations (e.g., Mysore and Knudsen, 2012), we suggest that LHb activity is a negative sigmoid function of BRS activation in response to reinforcing events (Figure 1). BRS activity could likewise be graphed as a negative sigmoid of LHb activity in response to punishing events. A simple linear function would be unsuitable because it does not capture that there are maximum and minimum limits on the brain's activity, nor does it capture the bidirectional nature of the relationship between the LHb and BRS.

Specifically, the function is sigmoid because when LHb neurons are activated during the prediction of a negative event, they inhibit the BRS and thereby disinhibit their own activity until they approach asymptotic levels of activation. Similarly, when BRS neurons are activated during the prediction of a positive event, they inhibit the LHb and thereby disinhibit their own activity until they approach asymptotic levels of activation. This would result in a tendency for the LHb and BRS to “switch” between bistable states whenever one component is perturbed by external stimulation, i.e., rewarding or punishing events. Thus, in terms of energy metabolism, a well-functioning LHb should be able to fluctuate between high and low energetic extremes, which might account for the large spontaneous variability (Gonzalez-Lima and Scheich, 1986) and the dramatic shifts in LHb metabolism relative to other brain regions that have been reported across multiple metabolic mapping studies (e.g., Gomita and Gallistel, 1982; Shumake et al., 2003).

What would happen if the LHb lost its capacity to dynamically shift its activational state in response to environmental changes? What if it became “stuck” in either a low or high energetic state? If the LHb were stuck in a low energetic state, the BRS would no longer be constrained by negative feedback. This could result in the auto-reinforcement of inappropriate behaviors and ideas that would normally be inhibited and discounted, possibly leading to disinhibition and delusions. Consistent with this conjecture, low LHb activity has been linked with schizophrenia (Lecourtier et al., 2004; Shepard et al., 2006). If the LHb were stuck in a high energetic state, the BRS would be indiscriminately constrained. This could result in the inappropriate discounting of events that would normally be regarded as reinforcing, with a consequential tendency toward apathy and anergia. Consistent with this conjecture, high LHb activity has been linked with depression (Morris et al., 1999; Roiser et al., 2009).

## Origins and consequences of persistently high LHb activity

Our own work suggests that persistently high LHb activity can be a consequence of genetic predisposition. Rats selectively bred for vulnerability to learned helplessness showed a striking *baseline* elevation of LHb metabolism compared to rats bred for resilience to learned helplessness, coincident with metabolic decreases throughout the BRS (Shumake et al., 2003). While learned helplessness results in a syndrome of behaviors resembling depression (Seligman, 1975), learned helplessness at its core represents a *failure to detect a negative reinforcement contingency* that would enable an organism to escape or avoid a stressor. Thus, we hypothesized that the association between greater LHb metabolism and vulnerability to learned helplessness exists because, if the LHb is stuck in a high energetic state, the response of the BRS to reinforcing events is chronically inhibited. This includes negative reinforcing events, such as terminating a foot shock, which activate the BRS in much the same way as positive reinforcing events (for review, see Ilango et al., 2012). Decreased salience of reinforcing events would impair the detection of stimulus-response contingences and lead to deficits in reinforcement learning.

We tested this hypothesis by implanting electrodes into the LHb and stimulating animals as they underwent training in a two-way active avoidance task (Shumake et al., 2010). Timing of LHb stimulation was coincident with and limited to the occurrence of each negative reinforcement event (in this case, crossing a hurdle to avoid a foot shock). As a consequence, learning of the avoidance response was impaired, demonstrating that inappropriate activity of the LHb during negative reinforcement can interfere with the acquisition of stimulus-response associations. Interestingly, this manipulation leads to a sustained deficit in avoidance responding that persists *even after LHb stimulation is discontinued*. Specifically, when five days of training are given with LHb stimulation, no behavioral improvement is seen after an additional five days of training without LHb stimulation (Shumake et al., 2010; Ilango et al., 2013). This suggests that repeated, sustained activation of the LHb not only serves to interrupt the concurrent behavior, but also serves to suppress future occurrences of the behavior. Similar effects of cumulative LHb stimulation on the suppression of positively reinforced eye saccades have also been reported (Matsumoto and Hikosaka, 2011).

Hypothetically, a cumulative function of LHb activations would have informative value across most motivated learning tasks in which the LHb is metabolically excited by failure and inhibited by success. For example, when learning a new task, intermittent failures provide information that, although improvement is needed, one is generally on the right track. However, repeated failures indicate that one is on the wrong track and that a more radical shift in strategy may be required. In this case, it would be adaptive for the LHb to have a mechanism by which it not only responds to immediate failure but also accumulates past failures. At some critical threshold of accumulated failure/frustration, we hypothesize that LHb activation becomes self-sustaining, providing a neural “give up” signal.

In normal circumstances, this signal should be temporary and adaptive. Abandoning an unsuccessful strategy makes way for learning a successful one, and, even if it doesn't, a variety of other reinforcing experiences in daily life should insure that the LHb does not become stuck in a high-activation state because of failure to obtain reinforcement in any one domain. However, for laboratory animals that are deprived of experiences of reinforcement outside of the experimental task, or for humans whose lives are dominated by chronic, unmanageable stress, repeated failure may result in “runaway” activation of the LHb that persists across situations and leads to symptoms of depression.

In summary, we hypothesize that the LHb is capable of entering into a self-sustaining high energetic state, which is reflected by large elevations in LHb resting metabolic activity. Laboratory experiments show that such elevations can be caused by genetics (Shumake et al., 2003) or by adverse environments (Caldecott-Hazard et al., 1988), but their ecological occurrence is likely a product of a gene × environment interaction. An individual born with the endophenotype of a highly energetic LHb should show relatively high sensitivity to punishment and relatively low sensitivity to reward. Over time, this could develop into an attentional bias toward negative stimuli and away from positive stimuli, a hallmark of the depressive phenotype (Disner et al., 2011).

However, endogenous inhibition of the BRS by the LHb is likely not absolute; rather, a hyperactive LHb is expected to raise the threshold for what is considered rewarding, such that only *intense* rewarding stimuli are capable of “flipping” the LHb-BRS metabolic “switch.” In the absence of adverse life events, this could manifest as a sensation-seeking personality, with the individual driven to pursue forms of reinforcement capable of overcoming LHb hyperinhibition, such as the intense positive reinforcement of using recreational drugs or the intense negative reinforcement of escaping from life-threatening situations. However, in the presence of adverse life events, LHb activity could be potentiated to such an extreme that no source of reinforcement is capable of overcoming its inertia, at which point the person enters a state of anhedonic withdrawal. There is indirect evidence to support this prediction: rats predisposed to helplessness in response to the severe stress of repeated foot shocks show baseline LHb hyperactivity (Shumake et al., 2003). However, before they are exposed to severe stress, these rats show increased risk-taking behavior and locomotor activation in response to novel environments (Shumake et al., 2005; Padilla et al., 2010), behaviors which are consistently linked to greater self-administration of DA drugs (Kabbaj et al., 2000).

## Conclusion: LHb metabolism as an inverse barometer of reinforcement sensitivity

The above scenario outlines *one* endophenotype that confers risk for depression; there are likely many others. The endophenotype we are describing is one of brief switches into an elevated mood state, but dominated by more protracted depressive states. In terms of clinical typology, this resembles bipolar II disorder, which may be the patient population most likely to show LHb effects. Supporting this, volumetric differences in the habenula are specifically associated with bipolar depression (Savitz et al., 2011). We have demonstrated how this endophenotype can be reduced to the dynamics of the LHb and its interactions with the BRS, but this should not be taken as an argument that the LHb is the only, or even the primary, locus of neuropathology. Rather, our argument is that baseline LHb hypermetabolism is a prominent neural sign of reinforcement insensitivity. The precise cellular-molecular causes of LHb hyperactivity could be distributed anywhere within the circuit (e.g., localized to the LHb itself, the regions from which it receives input, or in its projections to the midbrain). For example, potentiation of excitatory synapses onto LHb neurons projecting to the VTA (Li et al., 2011) provides one such cellular-molecular mechanism, but it is conceivable that the system could break down at other points in different individuals. Regardless, at the circuit level, LHb metabolism emerges as a common endpoint offering an index of trait reinforcement sensitivity and a potential therapeutic target for enhancing reinforcement sensitivity for patients in an anhedonic state.
